# Socioeconomic Determinants of Access to Medicines Among Romanian Patients with Chronic Diseases: A Cross-Sectional Study

**DOI:** 10.3390/healthcare14111453

**Published:** 2026-05-25

**Authors:** Corina Daniela Negrila, Luana-Maria Gherasie, Sebastian Mihai Armean, Petru Armean

**Affiliations:** 1 General Medicine, “Carol Davila” University of Medicine and Pharmacy, 050474 Bucharest, Romania; corina-daniela.negrila@drd.umfcd.ro (C.D.N.); petru.armean@umfcd.ro (P.A.); 2 “Prof. Dr. D. Hociotă” Institute of Phonoaudiology and Functional ENT Surgery, 050751 Bucharest, Romania; 3 Department of Pharmacology, Toxicology and Clinical Pharmacology, General Medicine, “Iuliu Hațieganu” University of Medicine and Pharmacy, 400012 Cluj-Napoca, Romania; sebastian.armean@umfcluj.ro; 4 ”Prof. Dr. Th. Burghele” Clinical Hospital, 050659 Bucharest, Romania

**Keywords:** access to medicines, pharmaceutical reimbursement, chronic disease, medicine affordability, health equity

## Abstract

**Background and Objectives:** Access to medicines is a fundamental determinant of health equity and a core pillar of universal health coverage, encompassing the timely availability, affordability, and appropriate use of essential medicines. Socioeconomic disparities may limit actual and timely access to pharmacological treatment, particularly in healthcare systems characterized by mixed public–private financing and significant out-of-pocket expenditures. This study aimed to evaluate socioeconomic determinants of access to medicines among Romanian patients with chronic diseases, focusing on income level, prescription reimbursement, perceived affordability, and substitution behavior during medicine shortages. **Materials and Methods:** A cross-sectional study was conducted between October and December 2024 using a structured online questionnaire administered to 200 adult patients diagnosed with cardiovascular diseases, diabetes mellitus, chronic hepatitis B and C, or oncological conditions, recruited at the “Prof. Dr. D. Hociotă” Institute of Phonoaudiology and Functional ENT Surgery, Bucharest, Romania. Associations between income and access-related variables were assessed using Spearman’s rank correlation coefficients with 95% confidence intervals. Binary logistic regression identified independent predictors of perceived difficulty in accessing medicines (*p* < 0.05). **Results:** Lower income was significantly associated with greater reliance on reimbursed prescriptions (r_s_ = −0.241, 95% CI: −0.37 to −0.10, *p* = 0.001) and fully reimbursed prescriptions (r_s_ = −0.305, 95% CI: −0.43 to −0.17, *p* < 0.001). Income was strongly correlated with perceived affordability of treatment (r_s_ = 0.601, 95% CI: 0.50–0.69, *p* < 0.001). In multivariate logistic regression analysis, income below 3000 RON/month (adjusted OR = 1.94, 95% CI: 1.05–3.58, *p* = 0.034) and insufficient affordability (adjusted OR = 4.12, 95% CI: 2.15–7.89, *p* < 0.001) were independently associated with perceived difficult access to treatment. Additionally, 80% of respondents reported purchasing substitute medicines when prescribed medicines were unavailable. **Conclusions:** This cross-sectional study indicates that socioeconomic status and perceived affordability are significant determinants of access to medicines among Romanian patients with chronic diseases attending a tertiary ENT centre. Financial vulnerability remains a major barrier despite existing reimbursement mechanisms. Policy interventions aimed at strengthening income-sensitive reimbursement strategies and ensuring consistent pharmaceutical availability may improve equitable access and therapeutic continuity.

## 1. Introduction

Access to medicines is a fundamental component of health system performance and a core pillar of universal health coverage, encompassing the timely availability, affordability, and appropriate use of essential medicines. Despite advances in pharmaceutical innovation and regulatory governance, disparities in access to medicines persist across healthcare systems globally. According to the World Health Organization framework, access to medicines is best understood through four interrelated dimensions: availability, accessibility, affordability, and acceptability [1,2]. The literature consistently emphasizes the multidimensional nature of access to medicines and the structural barriers affecting vulnerable populations [3]. Differences in pricing, reimbursement policies, and financing models can also significantly influence patients’ actual and timely access to medicines [4].

Globally, it has been estimated that a substantial proportion of the world’s population lacks reliable access to essential medicines, with this burden disproportionately affecting low- and middle-income settings [3,5]. Economic constraints remain among the most consistently reported determinants of restricted access to medicines. Cost-sharing mechanisms, including co-payments and partial reimbursement schemes, may reduce utilization of essential medicines, particularly among patients with chronic diseases requiring long-term treatment. Evidence from international health policy research indicates that increased out-of-pocket payments are associated with lower treatment adherence, delayed prescription filling, and poorer health outcomes [6,7]. Recent European data further confirm that unmet needs for access to medicines remain significantly associated with income gradients across EU member states [8,9].

Romania provides a relevant and timely setting for examining these challenges. Although the healthcare system is predominantly publicly financed, out-of-pocket expenditures remain substantial, particularly for outpatient pharmaceuticals [10,11]. Approximately 75% of medicines listed by the National Health Insurance House (Casa Nationala de Asigurari de Sanatate—CNAS) are eligible for reimbursement [12]. However, reimbursement levels, supply variability, and administrative constraints may influence real-world access to medicines. National pharmaceutical market data indicate fluctuations in medicine consumption and supply dynamics, potentially affecting therapeutic continuity [13].

Patients with chronic diseases—including cardiovascular diseases, diabetes mellitus, chronic viral hepatitis B and C, and oncological conditions—are particularly vulnerable to barriers in medicine access. Continuous pharmacological treatment is defined here as the ongoing, uninterrupted use of prescribed medicines for the management of a chronic condition over an extended period. When prescribed medicines are unavailable or unaffordable, patients may resort to therapeutic or generic substitution, delayed treatment, or self-medication—practices that may compromise therapeutic outcomes. Medication non-adherence related to financial constraints has been associated with increased healthcare utilization and worse clinical outcomes [6,14].

In Europe, socioeconomic inequalities in access to medicines represent a persistent challenge despite the existence of publicly financed healthcare systems. Pharmaceutical pricing regulations—defined as national policies governing the price-setting, reimbursement eligibility, and market authorization of medicinal products—vary substantially across member states and can significantly influence patients’ effective access. These disparities are particularly pronounced in Central and Eastern European healthcare systems, where household pharmaceutical expenditures remain relatively high compared with Western European countries [8,15].

Although international literature extensively documents macro-level determinants of access to medicines, patient-level empirical evidence examining the interaction among income, reimbursement mechanisms, perceived affordability, and substitution behavior within the Romanian healthcare context remains limited. Therefore, the objective of this study was to evaluate socioeconomic determinants of access to medicines among Romanian patients with chronic diseases, with a focus on the following self-reported independent variables: monthly income, use of reimbursed prescriptions, perceived affordability of treatment, and substitution behavior during medicine shortages.

We hypothesized that lower income and lower perceived affordability would be independently associated with higher odds of self-reported difficulty in accessing prescribed medicines.

## 2. Materials and Methods

### 2.1. Study Design and Reporting Standards

This research was designed as a cross-sectional observational study conducted between October and December 2024. The study was reported in accordance with the Strengthening the Reporting of Observational Studies in Epidemiology (STROBE) guidelines for cross-sectional studies [16]. The STROBE checklist is provided as Supplementary Material S1.

### 2.2. Study Setting and Participants

This study was conducted at the “Prof. Dr. D. Hociotă” Institute of Phonoaudiology and Functional ENT Surgery, Bucharest, Romania, a tertiary-level public institution. Participants were adult patients attending the institute who had a confirmed diagnosis of at least one chronic disease requiring continuous pharmacological treatment. Chronic diseases were selected based on their high epidemiological burden in Romania and their requirement for long-term pharmacological management [10,11]. The eligible conditions were: cardiovascular diseases (including arterial hypertension, coronary artery disease, heart failure, and atrial fibrillation), diabetes mellitus, chronic viral hepatitis B and C, and oncological diseases. All diagnoses were self-reported by participants and verified through a confirmatory question in the questionnaire. Participants were enrolled based on predefined inclusion and exclusion criteria, as detailed in Table 1.

A total of 214 individuals accessed the survey link. Of these, 205 provided electronic informed consent and initiated the questionnaire. Five responses were excluded due to incomplete mandatory items, and no confirmed duplicate submissions were identified. The final analytical sample comprised 200 complete responses (response rate: 93.5%). A non-probability convenience sampling strategy was applied.

Eligibility was confirmed through screening questions at survey entry, including: age ≥ 18 years, residence in Romania, self-reported diagnosis of one of the eligible chronic conditions, and ongoing pharmacological treatment.

### 2.3. Data Collection Instrument

Data were collected using a structured, self-administered online questionnaire developed in Romanian, based on prior literature on medicine access, reimbursement mechanisms, and substitution behavior [3,4,17]. Cultural and linguistic adaptation was performed by the research team to ensure comprehensibility for the Romanian patient population. The questionnaire comprised 28 closed-ended items grouped into the following domains: (1) sociodemographic variables (age, sex, residence, education level, employment status, marital status, and monthly income); (2) clinical variables (type of chronic disease); and (3) access-to-medicines variables (use of reimbursed prescriptions, use of fully reimbursed prescriptions, perceived affordability of treatment, perceived accessibility of medicines in pharmacies, and substitution behavior when prescribed medicines are unavailable). The complete questionnaire is provided as Supplementary Material S2.

The questionnaire was pilot-tested in 15 adult patients with chronic diseases to assess clarity, comprehensibility, and face validity. Minor linguistic adjustments were made following pilot testing to simplify wording, reduce ambiguity in income categorization, and improve clarity of reimbursement-related response options. No structural changes to the domains were required. Content validity was reviewed by two expert clinicians with experience in chronic disease management prior to pilot testing. Internal consistency of the access-to-medicines domain was assessed using Cronbach’s alpha (alpha = 0.74), indicating acceptable reliability.

The survey was administered electronically via the Google Forms platform. The form was configured to collect fully anonymous responses: no email addresses were collected, no Google account login was required, and no IP addresses were recorded. All participants had previously provided written informed consent for institutional data use in accordance with GDPR protocols at the time of admission to “Prof. Dr. D. Hociotă” Institute of Phonoaudiology and Functional ENT Surgery. Given the fully anonymous nature of data collection and the absence of any personally identifiable information, the platform configuration employed was considered consistent with ethical principles of privacy and confidentiality.

### 2.4. Operational Definitions and Variable Classification

Monthly income was categorized as <3000 RON/month (approximately 600 EUR) versus ≥3000 RON/month, a threshold approximating the national minimum wage in Romania during the study period and reflecting a policy-relevant distinction between financially vulnerable and relatively more secure households.

The primary outcome variable, perceived difficulty in accessing prescribed medicines, was assessed using the item: “How would you rate your overall access to the medicines prescribed for your chronic condition?” Responses were recorded on a 4-point Likert scale (1 = very easy, 2 = rather easy, 3 = rather difficult, 4 = very difficult) and dichotomized as easy access (very easy/rather easy = 0) and difficult access (rather difficult/very difficult = 1).

Perceived affordability of treatment was evaluated using the item: “To what extent is your monthly income sufficient to cover the cost of your prescribed treatment?” Responses were measured on a 4-point ordinal scale (1 = fully sufficient, 2 = mostly sufficient, 3 = mostly insufficient, 4 = completely insufficient) and categorized as sufficient (fully/mostly sufficient) versus insufficient (mostly/completely insufficient) for regression analyses.

Therapeutic substitution refers to the replacement of a prescribed medicine with a pharmacologically different but therapeutically equivalent alternative, while generic substitution refers to replacement with a bioequivalent product containing the same active ingredient. Both types are encompassed under the term “therapeutic or generic substitutes” in this study.

### 2.5. Bias and Quality Control

To limit selection bias, the survey was disseminated exclusively within the “Prof. Dr. D. Hociotă” Institute of Phonoaudiology and Functional ENT Surgery, targeting patients from both outpatient and inpatient settings across the institute’s clinical departments. To reduce the likelihood of duplicate entries, the platform was configured to allow a single submission per device session, and responses were manually screened for identical demographic and clinical patterns suggestive of duplication. To minimize information bias, all questions were formulated using standardized, neutral wording; key variables required for analysis were set as mandatory fields to prevent missing data, and clear response categories were provided to enhance consistency and reduce interpretation variability. Potential sources of bias include the self-reported nature of diagnoses and access-related assessments, as well as the convenience sampling strategy, which may limit the representativeness of the broader Romanian patient population.

### 2.6. Sample Size Considerations

The sample size (*n* = 200) was considered adequate to detect moderate correlation coefficients (r ≈ 0.30) with 80% statistical power at α = 0.05 [18]. Based on an anticipated prevalence of perceived difficulty in accessing medicines of approximately 35% (estimated from prior literature and pilot data), the expected number of outcome events was approximately 70. With seven candidate predictors included in the multivariate model, this corresponds to an events-per-variable (EPV) ratio of 10, satisfying the recommended minimum EPV threshold to ensure model stability and reduce the risk of overfitting [18,19].

### 2.7. Statistical Analysis

All analyses were conducted using IBM SPSS Statistics version 23 (IBM Corp., Armonk, NY, USA). Categorical variables were summarized using frequencies and percentages. Spearman’s rank correlation coefficients with 95% confidence intervals (calculated using Fisher’s z transformation) and exact two-tailed *p*-values were computed to assess associations between income and access-related variables, given the ordinal nature of the variables. Pearson correlation coefficients were additionally calculated as a sensitivity analysis and yielded consistent results in direction and magnitude. The full Spearman’s correlation matrix is provided in Supplementary Table S1. Binary logistic regression was performed to identify determinants of perceived difficulty in accessing medicines. Univariate models were first estimated; variables with *p* < 0.10 were included in the multivariate model. Age group, sex, residence, educational level, and chronic disease type were pre-specified as covariates regardless of univariate significance, to ensure appropriate confounding control. Multicollinearity was assessed using variance inflation factors (VIF > 5 considered indicative of potential multicollinearity). Model adequacy was evaluated using the Hosmer–Lemeshow goodness-of-fit test and Nagelkerke R^2^. The proportion of missing data was <5%; complete-case analysis was applied. The specification of the logistic regression model is detailed in Table 2.

### 2.8. Ethical Considerations

This study involved anonymous, questionnaire-based data collection without clinical intervention. Ethical review was conducted by the Ethics Committee of the “Prof. Dr. Dorin Hociotă” Institute of Phonoaudiology and Functional ENT Surgery (IFACF-ORL), which granted an ethical waiver (Reference No. 9930, dated 2 August 2024), prior to the initiation of data collection, in accordance with national regulations governing non-interventional observational research. The study was conducted in accordance with the Declaration of Helsinki [20]. All participants provided electronic informed consent prior to questionnaire completion.

## 3. Results

### 3.1. Participant Characteristics

A total of 200 respondents were included in the final analysis.

**Figure 1:**
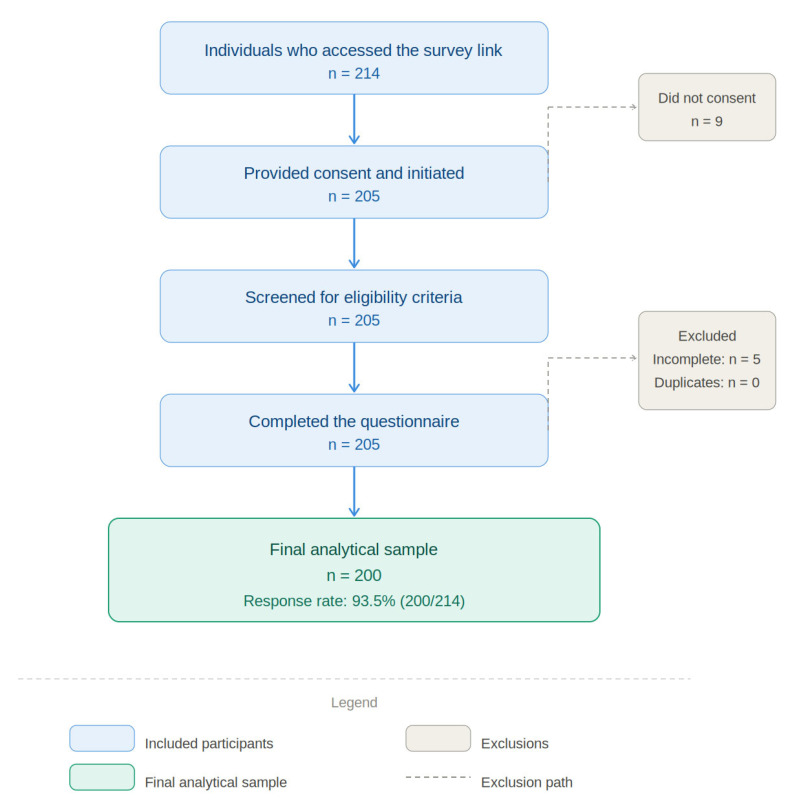
STROBE-compliant participant flow diagram illustrating survey recruitment, exclusions, and the final analytical sample (*n* = 200; response rate: 93.5%). Arrows indicate sequential steps from top to bottom.

A total of 214 individuals accessed the survey link. Of these, 205 provided electronic informed consent and initiated the questionnaire. Five responses were excluded due to incomplete mandatory items, and no confirmed duplicate submissions were identified after screening. The final analytical sample consisted of 200 complete responses. The recruitment and selection process of study participants is presented in Figure 1.

**Figure 2:**
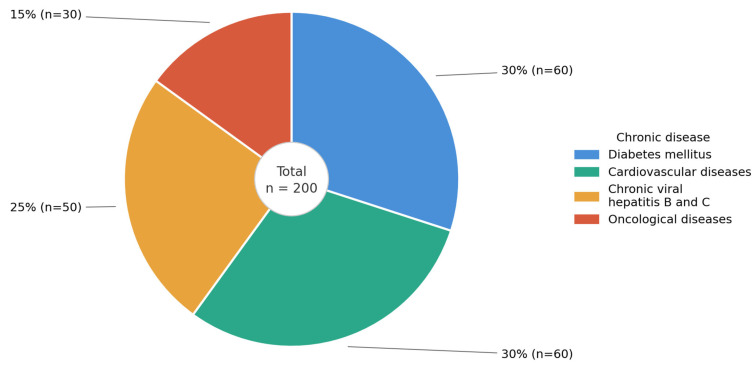
Distribution of chronic diseases among study participants (*n* = 200). Diabetes mellitus and cardiovascular diseases each accounted for 30% of cases (*n* = 60 each), followed by chronic viral hepatitis B and C (25%, *n* = 50) and oncological diseases (15%, *n* = 30).

The sociodemographic and clinical characteristics of the study population are summarized below. Of the 200 participants, 120 (60%) were female and 80 (40%) were male. The majority were aged 36–65 years (*n* = 120, 60%), followed by 18–35 years (*n* = 60, 30%) and over 65 years (*n* = 20, 10%). Most respondents resided in urban areas (*n* = 150, 75%). Regarding monthly income, 70 participants (35%) reported income below 3000 RON/month (~600 EUR), while 130 (65%) reported income ≥ 3000 RON/month. No statistically significant association was observed between income category and type of chronic disease (χ^2^ test, *p* = 0.41). Disease categories were mutually exclusive; participants were classified according to their primary chronic condition. The distribution of chronic diseases among study participants is illustrated in Figure 2, with diabetes mellitus and cardiovascular diseases each accounting for 30% of cases (*n* = 60 each), followed by chronic viral hepatitis B and C (*n* = 50, 25%) and oncological diseases (*n* = 30, 15%).

### 3.2. Correlation Analysis

Lower monthly income was significantly associated with greater reliance on reimbursed prescriptions (rs = −0.241, 95% CI: −0.37 to −0.10, *p* = 0.001) and fully reimbursed prescriptions (rs = −0.305, 95% CI: −0.43 to −0.17, *p* < 0.001). Income was strongly and positively correlated with perceived affordability of treatment (rs = 0.601, 95% CI: 0.50–0.69, *p* < 0.001), indicating that patients with lower incomes were substantially more likely to report insufficient affordability. Pearson correlation coefficients calculated as a sensitivity analysis confirmed the direction and magnitude of these associations. The full pairwise Spearman’s correlation matrix is provided in Supplementary Table S1.

### 3.3. Logistic Regression Analysis

The results of the unadjusted and adjusted logistic regression analyses are presented in Table 3. In the multivariate model, two variables were independently and significantly associated with perceived difficulty in accessing medicines. Income below 3000 RON/month (~600 EUR) was associated with nearly twice the odds of perceived difficulty in accessing medicines compared with higher income (adjusted OR = 1.94, 95% CI: 1.05–3.58, *p* = 0.034). Insufficient perceived affordability was the strongest independent predictor, with more than four times the odds of perceived difficulty in accessing medicines compared with sufficient affordability (adjusted OR = 4.12, 95% CI: 2.15–7.89, *p* < 0.001). Income and perceived affordability were each independently associated with the outcome; given the cross-sectional design and the absence of a formal mediation analysis, no causal or mediating relationship between these variables can be inferred. Rural residence showed a borderline association in the unadjusted model (unadjusted OR = 1.88, 95% CI: 1.01–3.52, *p* = 0.047) that did not reach statistical significance after adjustment (adjusted OR = 1.76, 95% CI: 0.94–3.29, *p* = 0.077). Sex, age group, education level, and chronic disease type were not significantly associated with the outcome in either unadjusted or adjusted analyses. The Hosmer–Lemeshow test indicated adequate model fit (*p* = 0.42), and the Nagelkerke R^2^ was 0.21.

**Figure 3:**
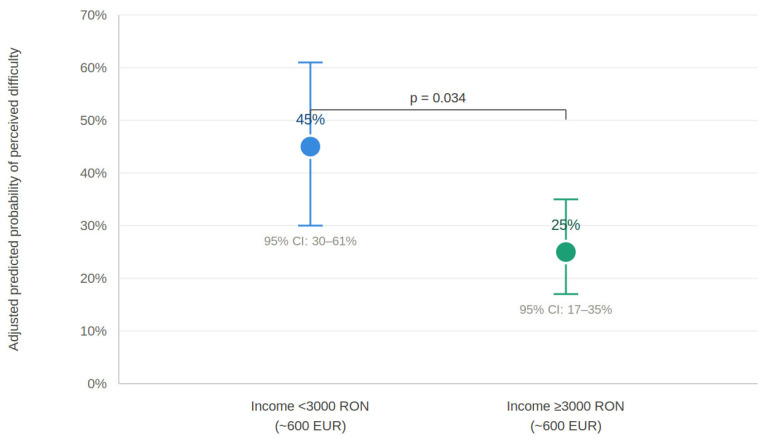
Adjusted predicted probabilities of perceived difficulty in accessing medicines by income category (*n* = 200). Patients with monthly income <3000 RON had a predicted probability of 45% compared with 25% among those with income ≥3000 RON.

**Figure 4:**
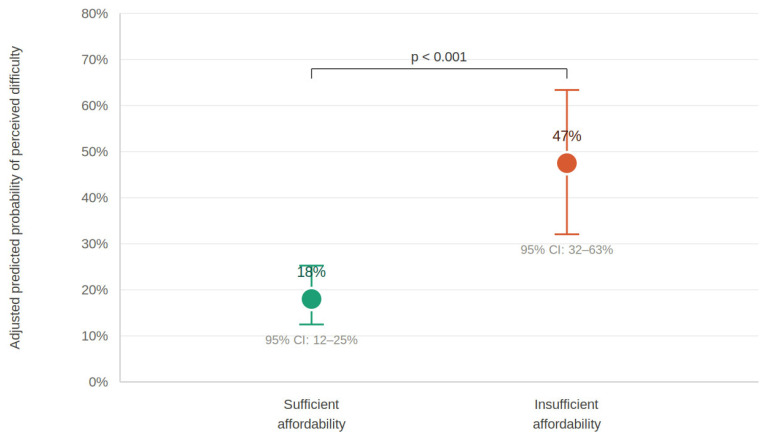
Adjusted predicted probabilities of perceived difficulty in accessing medicines by perceived affordability category (*n* = 200). Patients reporting insufficient affordability had a predicted probability of 47% compared with 18% among those reporting sufficient affordability.

The adjusted predicted probability of perceived difficulty in accessing medicines was 45% among patients with income below 3000 RON/month compared with 25% among those with higher income (Figure 3). Among patients reporting insufficient affordability, the predicted probability was 47%, compared with 18% among those with sufficient affordability (Figure 4).

### 3.4. Substitution Behavior

A total of 160 respondents (80%) reported purchasing therapeutic or generic substitutes when their prescribed medicines were unavailable. This finding was consistent across income categories and disease types, suggesting that medicine shortages represent a widespread challenge for Romanian patients with chronic conditions regardless of socioeconomic status.

Table 3 presents unadjusted and adjusted odds ratios (OR), 95% CI, and *p*-values for all candidate predictors.

## 4. Discussion

This cross-sectional study examined the socioeconomic determinants of access to medicines among Romanian patients with chronic diseases recruited at a single tertiary ENT centre. The findings indicate that lower income and insufficient perceived affordability are significant and independent determinants of perceived difficulty in accessing prescribed medicines, consistent with the study hypotheses and with existing international evidence [3,6,7].

The strong association between income and perceived affordability (rs = 0.601) suggests that affordability may represent a relevant proximal determinant linking socioeconomic status and access to medicines. Patients with lower incomes were substantially more likely to report that their monthly income was insufficient to cover treatment costs, which in turn was the strongest predictor of perceived access difficulties (adjusted OR = 4.12). This pattern is consistent with theoretical frameworks describing affordability as a proximal determinant of actual and timely access to medicines [1,2,21]. Although the reduction of the income effect after adjustment for affordability is compatible with a possible mediating role of affordability, the present cross-sectional design does not permit formal mediation testing, and any such interpretation should be regarded as exploratory.

Reimbursed prescriptions were significantly more prevalent among lower-income patients, reflecting appropriate utilization of public reimbursement mechanisms. However, the persistence of income-related access difficulties even after accounting for reimbursement use indicates that existing compensatory mechanisms are insufficient to fully offset financial barriers. Reimbursed prescriptions—defined here as prescriptions for which a co-payment is required but at a reduced rate—and fully reimbursed prescriptions—for which no co-payment is required—represent different tiers of financial protection within the Romanian CNAS system. Despite these mechanisms, patients with incomes below 3000 RON/month (~600 EUR) experienced nearly twice the odds of perceived access difficulty compared with higher-income patients, suggesting that co-payment levels and reimbursement eligibility criteria may still impose meaningful financial burdens on vulnerable households.

Medicine shortages represent an additional and growing dimension of access barriers in Romania and across European healthcare systems. In this study, 80% of respondents reported resorting to therapeutic or generic substitutes during medicine shortages, indicating that supply disruptions are highly prevalent and affect patients across income groups. Drug shortages have become a significant concern globally, driven by supply chain vulnerabilities, regulatory challenges, and economic pressures within the pharmaceutical market [22,23]. A comprehensive scoping review of nearly two decades of drug shortage literature emphasized the importance of proactive supply chain strategies, strengthened supplier coordination, and enhanced system preparedness to mitigate drug shortages and ensure continuity of care [24]. The COVID-19 pandemic further exposed critical fragilities in pharmaceutical supply chains, as demand surges and production disruptions led to severe shortages of essential medicines in many European countries, disproportionately affecting patients dependent on continuous pharmacological treatment. These experiences underline the necessity of robust national stockpiling strategies, diversified procurement policies, and pan-European coordination mechanisms to prevent future supply disruptions [25,26].

International experience offers important policy lessons for addressing access barriers to medicines. In the United States, public insurance programs such as Medicare and Medicaid have expanded access to medicines for elderly and low-income populations, respectively, with evidence suggesting reductions in medication non-adherence and improvements in chronic disease outcomes among beneficiaries. However, significant gaps remain, particularly in coverage of high-cost specialty medicines and for populations falling between eligibility thresholds. In Western Europe, countries such as France, Germany, and the Netherlands have implemented universal co-payment caps, income-graduated reimbursement tiers, and centralized pharmaceutical procurement mechanisms that have been associated with improved equity in access to medicines [27,28]. For Romania, a gradual transition toward income-sensitive reimbursement tiers, combined with investment in pharmaceutical supply chain resilience and expanded generic substitution policies, could represent feasible and effective policy levers to reduce socioeconomic disparities in access to medicines.

The finding that rural residence was associated with borderline higher odds of perceived access difficulties—though not statistically significant after adjustment—may reflect geographic disparities in pharmacy density and healthcare infrastructure, warranting further investigation in studies with adequate rural representation. The absence of significant associations for age, sex, and education in the adjusted model may reflect the dominant role of income and affordability as overarching determinants in this population, as well as sample size limitations for detecting smaller effects.

The present findings should be interpreted in the context of several limitations. The use of a non-probability convenience sample recruited within a single tertiary institution may limit the representativeness of the findings for the broader Romanian patient population, particularly for older individuals, patients with lower digital literacy, and those residing in rural or remote areas—groups that may face the greatest access barriers. The cross-sectional design precludes the establishment of causal relationships between socioeconomic variables and access to medicines. Self-reported data introduce the possibility of response bias, including social desirability effects, particularly regarding income declaration and reimbursement utilization. Disease diagnoses were self-reported and verified only through confirmatory questionnaire items rather than medical records, which may introduce misclassification. Finally, the dichotomization of income and affordability variables, while analytically appropriate given sample size constraints, may not fully capture the gradient nature of these constructs.

## 5. Conclusions

This cross-sectional study indicates that socioeconomic status and perceived affordability are significant determinants of access to medicines among Romanian patients with chronic diseases. Despite the existence of reimbursement mechanisms, financial vulnerability remains a substantial barrier to equitable access to medicines. Policy interventions aimed at strengthening income-sensitive reimbursement strategies, reducing pharmaceutical co-payments for vulnerable populations, and ensuring consistent pharmaceutical availability are likely to improve equitable access to medicines and therapeutic continuity for patients with chronic conditions in Romania. These findings should be interpreted with caution given the single-centre, non-probability sampling design, and are not directly generalisable to the broader Romanian population of patients with chronic diseases. Confirmation in larger, multi-centre studies with probability-based recruitment is warranted before drawing population-level inferences.

## Figures and Tables

**Table 1:** Inclusion and Exclusion Criteria.

Inclusion Criteria	Exclusion Criteria
Age ≥ 18 years	Acute conditions without chronic therapy
Confirmed chronic disease diagnosis	Incomplete questionnaires
Ongoing pharmacological treatment	Duplicate submissions
Completion of all key questionnaire items	
Provision of informed consent	

**Table 2:** Specification of the Logistic Regression Model.

Component	Description
**Type of analysis**	Binary logistic regression
**Objective**	To identify determinants of perceived difficult access to prescribed medicines
**Dependent variable**	Difficult access (1 = difficult, 0 = easy)
**Independent variables**	Monthly income
	Age group
	Sex
	Residence
	Educational level
	Chronic disease type
	Reimbursed prescription use
	Perceived affordability
**Model-building strategy**	Univariate models were first estimated. Variables with *p* < 0.10 were included in the multivariate model.
**Effect measures reported**	Odds ratios (OR)
	95% confidence intervals (95% CI)
	Exact *p*-values
**Model adequacy assessment**	Hosmer–Lemeshow goodness-of-fit test
	Nagelkerke R^2^
**Multicollinearity assessment**	Variance Inflation Factor (VIF); VIF > 5 considered indicative of potential multicollinearity
**Statistical significance threshold**	Two-tailed *p*-value < 0.05

**Table 3:** Unadjusted and adjusted logistic regression analysis of predictors of perceived difficulty in accessing prescribed medicines.

Variable	Category (Reference)	Unadjusted OR (95% CI)	*p*-Value	Adjusted OR (95% CI)	*p*-Value
Monthly income	<3000 RON (≥3000 RON)	2.31 (1.29–4.12)	0.005	1.94 (1.05–3.58)	0.034
Perceived affordability	Insufficient (Sufficient)	4.76 (2.54–8.91)	<0.001	4.12 (2.15–7.89)	<0.001
Residence	Rural (Urban)	1.88 (1.01–3.52)	0.047	1.76 (0.94–3.29)	0.077
Sex	Male (Female)	1.12 (0.63–1.99)	0.69	1.08 (0.58–2.01)	0.81
Age group	36–65 (18–35)	1.21 (0.63–2.34)	0.56	1.18 (0.59–2.37)	0.64
	>65 (18–35)	1.44 (0.55–3.75)	0.45	1.36 (0.50–3.71)	0.55
Education level	Secondary (University)	1.29 (0.67–2.48)	0.44	1.18 (0.60–2.34)	0.62
	Middle school (University)	1.61 (0.66–3.93)	0.29	1.42 (0.56–3.59)	0.46
Chronic disease type	Cardiovascular (Diabetes)	1.09 (0.55–2.17)	0.8	1.05 (0.51–2.15)	0.89
	Chronic hepatitis (Diabetes)	1.26 (0.61–2.62)	0.53	1.21 (0.57–2.58)	0.62
	Cancer (Diabetes)	1.34 (0.57–3.16)	0.5	1.28 (0.52–3.11)	0.59

## Data Availability

The data supporting the findings of this study are available from the corresponding author upon reasonable request. The dataset contains anonymized health-related information and is not publicly available due to privacy and ethical considerations.

## Supplementary Materials

The following supporting information can be downloaded at: https://www.mdpi.com/article/10.3390/healthcare14111453/s1, Material S1: STROBE checklist for cross-sectional studies; Material S2: Full study questionnaire (Romanian original and English translation); Table S1: Comprehensive Spearman’s rank correlation matrix including all pairwise correlations between socioeconomic and access-related variables, with 95% confidence intervals. Reference [29] is cited in the supplementary materials.

## Abbreviations

The following abbreviations are used in this manuscript:

CI	Confidence Interval
CNAS	Casa Națională de Asigurări de Sănătate
GDPR	General Data Protection Regulation
OR	Odds Ratio
RON	Romanian Leu
SPSS	Statistical Package for the Social Sciences
STROBE	Strengthening the Reporting of Observational Studies in Epidemiology
VIF	Variance Inflation Factor
