# Supplementary material for: Socioeconomic Determinants of Access to Medicines Among Romanian Patients with Chronic Diseases: A Cross-Sectional Study
Source: Healthcare (Basel). 2026 May 25;14(11):1453. doi: 10.3390/healthcare14111453 (PMC13256319; doi:10.3390/healthcare14111453)
Supplement: Supplementary file 1 [file healthcare-14-01453-s001.zip › Supplementary Material S1 STROBE Checklist.pdf]

Supplementary Material S1

STROBE Statement — Checklist of items that should be included in reports of cross-sectional studies

Item No.	Recommendation	Page / Section in manuscript
**TITLE AND ABSTRACT**		
1	(a) Indicate the study's design with a commonly used term in the title or the abstract	Title (p. 1); Abstract (p. 1, Materials and Methods)
	(b) Provide in the abstract an informative and balanced summary of what was done and what was found	Abstract (p. 1)
**INTRODUCTION**		
2	**Background/rationale:** Explain the scientific background and rationale for the investigation being reported	Introduction, paragraphs 1–4 (Section 1)
3	**Objectives:** State specific objectives, including any pre-specified hypotheses	Introduction, final paragraph (Section 1, hypothesis statement)
**METHODS**		
4	**Study design:** Present key elements of study design early in the paper	Section 2.1 (Study Design and Reporting Standards)
5	**Setting:** Describe the setting, locations, and relevant dates, including periods of recruitment, exposure, follow-up, and data collection	Section 2.1 and 2.2 (October–December 2024; IFACF-ORL Bucharest)
6	**Participants:** (a) Cross-sectional study—Give the eligibility criteria, and the sources and methods of selection of participants	Section 2.2 (Study Setting and Participants); Table 1 (Inclusion/Exclusion criteria)
7	**Variables:** Clearly define all outcomes, exposures, predictors, potential confounders, and effect modifiers. Give diagnostic criteria, if applicable	Section 2.4 (Operational Definitions and Variable Classification)
8*	**Data sources/measurement:** For each variable of interest, give sources of data and details of methods of assessment (measurement). Describe comparability of assessment methods if there is more than one group	Section 2.3 (Data Collection Instrument); Section 2.4
9	**Bias:** Describe any efforts to address potential sources of bias	Section 2.5 (Bias and quality control)
10	**Study size:** Explain how the study size was arrived at	Section 2.6 (Sample Size Considerations); reference [16] (Peduzzi 1996, EPV ≥10 rule)
11	**Quantitative variables:** Explain how quantitative variables were handled in the analyses. If applicable, describe which groupings were chosen, and why	Section 2.4 (income dichotomization at 3000 RON); Section 2.7 (Statistical Analysis)
12	**Statistical methods:** (a) Describe all statistical methods, including those used to control for confounding	Section 2.7 (Statistical Analysis); Table 2 (Logistic Regression Specification)
	(b) Describe any methods used to examine subgroups and interactions	Section 2.7 (no subgroup analyses pre-specified; covariate-adjusted models reported)
	(c) Explain how missing data were addressed	Section 2.7 (<5% missing; complete-case analysis)
	(d) Cross-sectional study—If applicable, describe analytical methods taking account of sampling strategy	Section 2.7 and Section 2.5 (non-probability convenience sample; limitations acknowledged)
	(e) Describe any sensitivity analyses	Section 2.7 (Pearson correlation as sensitivity analysis to primary Spearman); Supplementary Table S1

Item No.	Recommendation	Page / Section in manuscript
**RESULTS**		
**13***	**Participants:** (a) Report numbers of individuals at each stage of study—e.g., numbers potentially eligible, examined for eligibility, confirmed eligible, included in the study, completing follow-up, and analysed	Section 3.1; Figure 1 (STROBE flow diagram)
	(b) Give reasons for non-participation at each stage	Section 3.1 and Figure 1 (5 incomplete responses excluded; no duplicates)
	(c) Consider use of a flow diagram	Figure 1 (provided)
**14***	**Descriptive data:** (a) Give characteristics of study participants (e.g., demographic, clinical, social) and information on exposures and potential confounders	Section 3.1 (sociodemographic and clinical characteristics)
	(b) Indicate number of participants with missing data for each variable of interest	Section 2.7 (<5% missing across all variables)
**15***	**Outcome data:** Cross-sectional study—Report numbers of outcome events or summary measures	Section 3.1, 3.2, 3.3 and 3.4; Figure 2; Table 3
**16**	**Main results:** (a) Give unadjusted estimates and, if applicable, confounder-adjusted estimates and their precision (e.g., 95% confidence interval). Make clear which confounders were adjusted for and why they were included	Section 3.3; Table 3 (unadjusted and adjusted ORs with 95% CI)
	(b) Report category boundaries when continuous variables were categorized	Section 2.4 (income <3000 RON vs ≥3000 RON)
	(c) If relevant, consider translating estimates of relative risk into absolute risk for a meaningful time period	Section 3.3 (adjusted predicted probabilities reported in addition to OR — Figures 3 and 4)
**17**	**Other analyses:** Report other analyses done—e.g., analyses of subgroups and interactions, and sensitivity analyses	Section 3.2 (Pearson sensitivity analysis); Supplementary Table S1
**DISCUSSION**		
**18**	**Key results:** Summarise key results with reference to study objectives	Section 4 (Discussion, opening paragraph)
**19**	**Limitations:** Discuss limitations of the study, taking into account sources of potential bias or imprecision. Discuss both direction and magnitude of any potential bias	Section 4 (Limitations paragraph)
**20**	**Interpretation:** Give a cautious overall interpretation of results considering objectives, limitations, multiplicity of analyses, results from similar studies, and other relevant evidence	Section 4 (Discussion paragraphs 2–5)
**21**	**Generalisability:** Discuss the generalisability (external validity) of the study results	Section 4 (Limitations) and Section 5 (Conclusions, closing limitation paragraph)
**OTHER INFORMATION**		
**22**	**Funding:** Give the source of funding and the role of the funders for the present study and, if applicable, for the original study on which the present article is based	Funding statement (back matter): "This research received no external funding."

Reference: von Elm E, Altman DG, Egger M, Pocock SJ, Gøtzsche PC, Vandenbroucke JP. The Strengthening the Reporting of Observational Studies in Epidemiology (STROBE) statement: guidelines for reporting observational studies. *Lancet* 2007;370(9596):1453–1457. https://doi.org/10.1016/S0140-6736(07)61602-X
