# Supplementary material for: Socioeconomic Determinants of Access to Medicines Among Romanian Patients with Chronic Diseases: A Cross-Sectional Study
Source: Healthcare (Basel). 2026 May 25;14(11):1453. doi: 10.3390/healthcare14111453 (PMC13256319; doi:10.3390/healthcare14111453)
Supplement: Supplementary file 1 [file healthcare-14-01453-s001.zip › Supplementary Material S2 Questionnaire.pdf]

Supplementary Material S2

Study Questionnaire — Romanian original and English translation

Screening și consimțământ informat / Screening and informed consent

Versiunea în limba română	English version
**QS1.** Aveți cel puțin 18 ani împliniți?<br>☐ Da<br>☐ Nu (nu sunteți eligibil/ă)	**QS1.** Are you at least 18 years old?<br>☐ Yes<br>☐ No (not eligible)
**QS2.** Sunteți rezident/ă în România?<br>☐ Da<br>☐ Nu (nu sunteți eligibil/ă)	**QS2.** Are you a resident in Romania?<br>☐ Yes<br>☐ No (not eligible)
**QS3.** Aveți un diagnostic confirmat de cel puțin una dintre următoarele boli cronice: boală cardiovasculară, diabet zaharat, hepatită cronică virală B sau C, sau o afecțiune oncologică?<br>☐ Da<br>☐ Nu (nu sunteți eligibil/ă)	**QS3.** Do you have a confirmed diagnosis of at least one of the following chronic diseases: cardiovascular disease, diabetes mellitus, chronic viral hepatitis B or C, or an oncological condition?<br>☐ Yes<br>☐ No (not eligible)
**QS4.** Urmați în prezent un tratament medicamentos continuu pentru această afecțiune cronică?<br>☐ Da<br>☐ Nu (nu sunteți eligibil/ă)	**QS4.** Are you currently receiving ongoing pharmacological treatment for this chronic condition?<br>☐ Yes<br>☐ No (not eligible)
**QS5.** Sunt de acord să particip voluntar la acest studiu și înțeleg că răspunsurile mele vor fi colectate anonim, fără date personale identificabile, și utilizate exclusiv în scop de cercetare.<br>☐ Da, sunt de acord (continuă chestionarul)<br>☐ Nu sunt de acord (nu se completează chestionarul)	**QS5.** I voluntarily agree to participate in this study and I understand that my responses will be collected anonymously, without identifiable personal data, and used exclusively for research purposes.<br>☐ Yes, I agree (continue questionnaire)<br>☐ No, I do not agree (questionnaire not completed)

Secțiunea 1. Variabile sociodemografice / Section 1. Sociodemographic variables

Versiunea în limba română	English version
**Q1.** Vârsta dumneavoastră (în ani împliniți):<br>☐ 18–35 ani<br>☐ 36–65 ani<br>☐ Peste 65 de ani	**Q1.** Your age (completed years):<br>☐ 18–35 years<br>☐ 36–65 years<br>☐ Over 65 years
**Q2.** Sexul:	**Q2.** Sex:

Versiunea în limba română	English version
☐ Feminin<br>☐ Masculin	☐ Female<br>☐ Male
**Q3.** Mediul de rezidență:<br>☐ Urban<br>☐ Rural	**Q3.** Area of residence:<br>☐ Urban<br>☐ Rural
**Q4.** Ultimul nivel de studii absolvit:<br>☐ Studii primare/gimnaziale (≤8 clase)<br>☐ Studii liceale/postliceale (secundar)<br>☐ Studii universitare sau postuniversitare	**Q4.** Highest level of education completed:<br>☐ Primary/middle school (≤8 grades)<br>☐ High school/post-high school (secondary)<br>☐ University or postgraduate
**Q5.** Statutul ocupațional actual:<br>☐ Angajat/ă cu normă întreagă<br>☐ Angajat/ă cu normă parțială<br>☐ Independent/PFA<br>☐ Pensionar/ă<br>☐ Șomer/ă<br>☐ Altul	**Q5.** Current employment status:<br>☐ Full-time employed<br>☐ Part-time employed<br>☐ Self-employed<br>☐ Retired<br>☐ Unemployed<br>☐ Other
**Q6.** Statutul marital:<br>☐ Necăsătorit/ă<br>☐ Căsătorit/ă sau în uniune consensuală<br>☐ Divorțat/ă sau separat/ă<br>☐ Văduv/ă	**Q6.** Marital status:<br>☐ Single<br>☐ Married or in a consensual union<br>☐ Divorced or separated<br>☐ Widowed
**Q7.** Venitul lunar net al gospodăriei dumneavoastră:<br>☐ Sub 3000 RON/lună (≈ 600 EUR)<br>☐ 3000 RON sau peste pe lună (≥ 600 EUR)	**Q7.** Your monthly net household income:<br>☐ Below 3000 RON/month (≈ 600 EUR)<br>☐ 3000 RON or above per month (≥ 600 EUR)
**Q8.** Numărul total de persoane din gospodărie (incluzând respondentul):<br>☐ 1 persoană<br>☐ 2 persoane<br>☐ 3–4 persoane<br>☐ ≥ 5 persoane	**Q8.** Total number of persons in the household (including respondent):<br>☐ 1 person<br>☐ 2 persons<br>☐ 3–4 persons<br>☐ ≥ 5 persons
**Q9.** Aveți asigurare medicală prin Casa Națională de Asigurări de Sănătate (CNAS)?<br>☐ Da<br>☐ Nu<br>☐ Nu știu	**Q9.** Do you have health insurance through the National Health Insurance House (CNAS)?<br>☐ Yes<br>☐ No<br>☐ I don't know

Secțiunea 2. Variabile clinice / Section 2. Clinical variables

Versiunea în limba română	English version
**Q10.** Care este afecțiunea cronică principală pentru care urmați tratament continuu?<br>☐ Boală cardiovasculară (ex. hipertensiune, boală coronariană, insuficiență cardiacă, fibrilație atrială)<br>☐ Diabet zaharat<br>☐ Hepatită cronică virală B sau C<br>☐ Afecțiune oncologică<br>☐ Altă afecțiune cronică	**Q10.** What is the main chronic condition for which you are receiving continuous treatment?<br>☐ Cardiovascular disease (e.g., hypertension, coronary artery disease, heart failure, atrial fibrillation)<br>☐ Diabetes mellitus<br>☐ Chronic viral hepatitis B or C<br>☐ Oncological condition<br>☐ Other chronic condition
**Q11.** De câți ani urmați tratament pentru această afecțiune cronică?<br>☐ Sub 1 an<br>☐ 1–5 ani<br>☐ 6–10 ani<br>☐ Peste 10 ani	**Q11.** For how many years have you been receiving treatment for this chronic condition?<br>☐ Less than 1 year<br>☐ 1–5 years<br>☐ 6–10 years<br>☐ More than 10 years
**Q12.** Câte medicamente diferite primiți pe rețetă în mod curent pentru această afecțiune?<br>☐ 1 medicament<br>☐ 2 medicamente<br>☐ 3 medicamente<br>☐ 4 sau mai multe medicamente	**Q12.** How many different prescription medicines do you currently receive for this condition?<br>☐ 1 medicine<br>☐ 2 medicines<br>☐ 3 medicines<br>☐ 4 or more medicines
**Q13.** Mai aveți și alte boli cronice (comorbidități) care necesită tratament?<br>☐ Da<br>☐ Nu	**Q13.** Do you have other chronic diseases (comorbidities) requiring treatment?<br>☐ Yes<br>☐ No
**Q14.** În general, cum ați descrie starea dumneavoastră de sănătate în ultimul an?<br>☐ Foarte bună<br>☐ Bună<br>☐ Satisfăcătoare<br>☐ Slabă<br>☐ Foarte slabă	**Q14.** Overall, how would you describe your health status during the last year?<br>☐ Very good<br>☐ Good<br>☐ Fair<br>☐ Poor<br>☐ Very poor

Secțiunea 3. Accesul la medicamente / Section 3. Access to medicines

Versiunea în limba română	English version
**Q15.** Cât de des utilizați rețete compensate (parțial gratuite) pentru medicamentele dumneavoastră?<br>☐ Întotdeauna<br>☐ Frecvent<br>☐ Uneori<br>☐ Rareori<br>☐ Niciodată	**Q15.** How often do you use reimbursed prescriptions (partially free) for your medicines?<br>☐ Always<br>☐ Frequently<br>☐ Sometimes<br>☐ Rarely<br>☐ Never
**Q16.** Cât de des utilizați rețete gratuite (compensate integral) pentru medicamentele dumneavoastră?<br>☐ Întotdeauna<br>☐ Frecvent<br>☐ Uneori<br>☐ Rareori<br>☐ Niciodată	**Q16.** How often do you use fully reimbursed prescriptions (free) for your medicines?<br>☐ Always<br>☐ Frequently<br>☐ Sometimes<br>☐ Rarely<br>☐ Never
**Q17.** În ce măsură venitul dumneavoastră lunar este suficient pentru a acoperi costul tratamentului prescris?<br>☐ Complet suficient<br>☐ În mare măsură suficient<br>☐ În mică măsură suficient (insuficient)<br>☐ Deloc suficient (complet insuficient)	**Q17.** To what extent is your monthly income sufficient to cover the cost of your prescribed treatment?<br>☐ Fully sufficient<br>☐ Mostly sufficient<br>☐ Mostly insufficient<br>☐ Completely insufficient
**Q18.** Cum apreciați disponibilitatea medicamentelor prescrise în farmaciile la care apelați în mod obișnuit?<br>☐ Foarte bună – aproape întotdeauna disponibile<br>☐ Bună – de regulă disponibile<br>☐ Variabilă – uneori indisponibile<br>☐ Slabă – frecvent indisponibile	**Q18.** How would you rate the availability of your prescribed medicines in the pharmacies you usually use?<br>☐ Very good — almost always available<br>☐ Good — usually available<br>☐ Variable — sometimes unavailable<br>☐ Poor — frequently unavailable
**Q19.** Cum ați evalua, în ansamblu, accesul dumneavoastră la medicamentele prescrise pentru afecțiunea cronică?<br>☐ Foarte ușor<br>☐ Destul de ușor<br>☐ Destul de dificil<br>☐ Foarte dificil	**Q19.** How would you rate your overall access to the medicines prescribed for your chronic condition?<br>☐ Very easy<br>☐ Rather easy<br>☐ Rather difficult<br>☐ Very difficult
**Q20.** În ultimele 12 luni, vi s-a întâmplat să nu puteți achiziționa un medicament prescris din motive financiare?<br>☐ Niciodată<br>☐ O dată<br>☐ De 2–3 ori<br>☐ De 4 sau mai multe ori	**Q20.** In the last 12 months, have you been unable to purchase a prescribed medicine for financial reasons?<br>☐ Never<br>☐ Once<br>☐ 2–3 times<br>☐ 4 or more times

Versiunea în limba română	English version
**Q21.** În ultimele 12 luni, vi s-a întâmplat să întârziați sau să întrerupeți un tratament prescris din motive financiare?<br>☐ Niciodată<br>☐ Rareori<br>☐ Uneori<br>☐ Frecvent	**Q21.** In the last 12 months, have you delayed or interrupted a prescribed treatment for financial reasons?<br>☐ Never<br>☐ Rarely<br>☐ Sometimes<br>☐ Frequently
**Q22.** În ultimele 12 luni, vi s-a întâmplat ca un medicament prescris să nu fie disponibil în farmaciile la care v-ați adresat?<br>☐ Niciodată<br>☐ O dată<br>☐ De 2–3 ori<br>☐ De 4 sau mai multe ori	**Q22.** In the last 12 months, has a prescribed medicine been unavailable in the pharmacies you visited?<br>☐ Never<br>☐ Once<br>☐ 2–3 times<br>☐ 4 or more times
**Q23.** Atunci când medicamentul prescris nu a fost disponibil, ați recurs la un substitut terapeutic sau generic?<br>☐ Da<br>☐ Nu<br>☐ Nu a fost cazul	**Q23.** When the prescribed medicine was unavailable, did you resort to a therapeutic or generic substitute?<br>☐ Yes<br>☐ No<br>☐ Not applicable
**Q24.** Dacă da, cine v-a recomandat substitutul?<br>☐ Medicul curant<br>☐ Farmacistul<br>☐ Decizie proprie<br>☐ Altă persoană / nu se aplică	**Q24.** If yes, who recommended the substitute?<br>☐ Treating physician<br>☐ Pharmacist<br>☐ My own decision<br>☐ Other person / not applicable
**Q25.** Ați achiziționat vreodată medicamente din afara României (de ex. din alte țări UE) din cauza indisponibilității în țară?<br>☐ Da<br>☐ Nu	**Q25.** Have you ever purchased medicines from outside Romania (e.g., from other EU countries) due to unavailability domestically?<br>☐ Yes<br>☐ No
**Q26.** Cum apreciați costul lunar suportat din buzunar (out-of-pocket) pentru tratamentul afecțiunii cronice?<br>☐ Sub 100 RON<br>☐ 100–300 RON<br>☐ 301–500 RON<br>☐ Peste 500 RON	**Q26.** How would you rate your monthly out-of-pocket expenditure for the chronic disease treatment?<br>☐ Below 100 RON<br>☐ 100–300 RON<br>☐ 301–500 RON<br>☐ Above 500 RON
**Q27.** Cât de mulțumit/ă sunteți, în general, de modul în care sistemul național de asigurări de sănătate (CNAS) acoperă costul tratamentului dumneavoastră?<br>☐ Foarte mulțumit/ă<br>☐ Mulțumit/ă	**Q27.** How satisfied are you, overall, with how the national health insurance system (CNAS) covers the cost of your treatment?<br>☐ Very satisfied<br>☐ Satisfied

Versiunea în limba română	English version
☐ Nici mulțumit/ă, nici nemulțumit/ă<br>☐ Nemulțumit/ă<br>☐ Foarte nemulțumit/ă	☐ Neither satisfied nor dissatisfied<br>☐ Dissatisfied<br>☐ Very dissatisfied
**Q28.** În opinia dumneavoastră, care ar fi cea mai importantă măsură pentru îmbunătățirea accesului la medicamente pentru pacienții cu boli cronice din România?<br>☐ Reducerea costurilor de coplată<br>☐ Lărgirea listei de medicamente compensate<br>☐ Asigurarea continuității în farmacii (reducerea lipsurilor)<br>☐ Sprijin financiar specific pentru pacienții cu venituri reduse<br>☐ Alta	**Q28.** In your opinion, what would be the most important measure to improve access to medicines for patients with chronic diseases in Romania?<br>☐ Reducing co-payment costs<br>☐ Expanding the list of reimbursed medicines<br>☐ Ensuring continuous availability in pharmacies (reducing shortages)<br>☐ Specific financial support for low-income patients<br>☐ Other

Notă privind administrarea: *Chestionarul a fost administrat electronic, prin platforma Google Forms, în mod complet anonim (fără colectare de adrese de e-mail, fără autentificare cu cont Google și fără înregistrare de adrese IP). Adaptarea culturală și lingvistică a fost realizată de echipa de cercetare; testarea pilot a fost efectuată pe 15 pacienți adulți cu boli cronice. Consistența internă a domeniului accesului la medicamente a fost evaluată prin coeficientul Cronbach alpha (α = 0.74).*

Administration note: *The questionnaire was administered electronically via Google Forms, in fully anonymous mode (no email collection, no Google account login required, no IP address recording). Cultural and linguistic adaptation was performed by the research team; pilot testing was performed on 15 adult patients with chronic diseases. Internal consistency of the access-to-medicines domain was assessed using Cronbach's alpha (α = 0.74).*
