# Supplementary material for: Socioeconomic Determinants of Access to Medicines Among Romanian Patients with Chronic Diseases: A Cross-Sectional Study
Source: Healthcare (Basel). 2026 May 25;14(11):1453. doi: 10.3390/healthcare14111453 (PMC13256319; doi:10.3390/healthcare14111453)
Supplement: Supplementary file 1 [file healthcare-14-01453-s001.zip › Supplementary Table S1 Spearman Correlation Matrix.pdf]

Supplementary Table S1

Spearman rank correlation coefficients between socioeconomic and access-related variables

Spearman rank correlation coefficients (rs) with 95% confidence intervals (calculated using Fisher's z transformation) and exact two-tailed p-values for the pairwise associations between income and access-related variables (n = 200).

Variable pair	rs	95% CI	p-value	Interpretation
Monthly income vs. use of reimbursed prescriptions	−0.241	−0.37 to −0.10	0.001	Weak negative
Monthly income vs. use of fully reimbursed prescriptions	−0.305	−0.43 to −0.17	<0.001	Weak–moderate negative
Monthly income vs. perceived affordability of treatment	0.601	0.50 to 0.69	<0.001	Moderate–strong positive
Monthly income vs. perceived difficulty in accessing medicines	−0.278	−0.40 to −0.15	<0.001	Weak negative
Perceived affordability vs. perceived difficulty in accessing medicines	0.512	0.40 to 0.61	<0.001	Moderate positive

Abbreviations: *rs* = Spearman rank correlation coefficient; CI = confidence interval.

Notes. 95% CI calculated using Fisher's z transformation. Negative values indicate that higher income is associated with lower reliance on reimbursed/fully reimbursed prescriptions and lower perceived difficulty in accessing medicines. Positive values indicate that higher income is associated with better perceived affordability.

Correlation strength interpretation [29]: |*rs*| < 0.30 = weak; 0.30–0.50 = moderate; > 0.50 = strong.

Sensitivity analysis. Results are consistent in direction and magnitude with the Pearson correlation analysis used as a sensitivity check (see main manuscript, Sections 2.7 and 3.2). Statistical significance was maintained for all reported associations.

Software. All analyses were conducted using IBM SPSS Statistics version 23 (IBM Corp., Armonk, NY, USA).
